# Effects of L-Citrulline Supplementation on Endothelial Function and Blood Pressure in Hypertensive Postmenopausal Women

**DOI:** 10.3390/nu14204396

**Published:** 2022-10-20

**Authors:** Arun Maharaj, Stephen M. Fischer, Katherine N. Dillon, Yejin Kang, Mauricio A. Martinez, Arturo Figueroa

**Affiliations:** Department of Kinesiology and Sport Management, Texas Tech University, Lubbock, TX 79409, USA

**Keywords:** citrulline, endothelial function, arterial stiffness, aortic blood pressure, hypertension, postmenopausal women

## Abstract

Aging and menopause are associated with decreased nitric oxide bioavailability due to reduced L-arginine (L-ARG) levels contributing to endothelial dysfunction (ED). ED precedes arterial stiffness and hypertension development, a major risk factor for cardiovascular disease. This study investigated the effects of L-citrulline (L-CIT) on endothelial function, aortic stiffness, and resting brachial and aortic blood pressures (BP) in hypertensive postmenopausal women. Twenty-five postmenopausal women were randomized to 4 weeks of L-CIT (10 g) or placebo (PL). Serum L-ARG, brachial artery flow-mediated dilation (FMD), aortic stiffness (carotid-femoral pulse wave velocity, cfPWV), and resting brachial and aortic BP were assessed at 0 and 4 weeks. L-CIT supplementation increased L-ARG levels (Δ13 ± 2 vs. Δ−2 ± 2 µmol/L, *p* < 0.01) and FMD (Δ1.4 ± 2.0% vs. Δ−0.5 ± 1.7%, *p* = 0.03) compared to PL. Resting aortic diastolic BP (Δ−2 ± 4 vs. Δ2 ± 5 mmHg, *p* = 0.01) and mean arterial pressure (Δ−2 ± 4 vs. Δ2 ± 6 mmHg, *p* = 0.04) were significantly decreased after 4 weeks of L-CIT compared to PL. Although not statistically significant (*p* = 0.07), cfPWV decreased after L-CIT supplementation by ~0.66 m/s. These findings suggest that L-CIT supplementation improves endothelial function and aortic BP via increased L-ARG availability.

## 1. Introduction

Aging is associated with a steady rise in incident cardiovascular disease (CVD) in women [1]. Approximately, 75% and 78% of postmenopausal women aged 65 to 75 and older than 75 years in the United States are hypertensive [2,3]. Women with hypertension have higher prevalence of cardiometabolic risk factors, such as abdominal obesity and hypercholesterolemia, and less control of blood pressure with antihypertensive therapy than men [4]. A greater aortic systolic blood pressure may contribute to the higher risk of heart failure with preserved ejection fraction in women than men [5].

Endothelial function is associated with nitric oxide (NO) availability for vasodilation, blood pressure and blood flow regulation, and vascular health [6]. Brachial artery flow-mediated dilation (FMD), the non-invasive gold standard method for endothelial function assessment [7], decreases across the menopausal transition in healthy women [8]. The decline in brachial FMD begins during the perimenopausal period and progresses further after the menopause [8]. A steeper rate of endothelial dysfunction occurs in postmenopausal women than in men [9]. A mechanism of endothelial dysfunction in postmenopausal women is reduced availability of L-arginine (L-ARG), the substrate for NO synthesis [10]. This L-ARG deficiency may be associated with increased catabolism of L-ARG to ornithine by the enzyme arginase [10]. Reduced NO production may partially explain the increased prevalence of hypertension in older women [11,12,13]. Once endothelial dysfunction is established, structural changes in the vascular wall contribute to increase arterial stiffness [14,15], which is assessed by pulse wave velocity (PWV). Endothelial dysfunction [16] and aortic stiffness [17,18,19] are independent predictors for the development of hypertension and cardiovascular disease [20,21,22,23,24,25].

The amino acids L-ARG and L-citrulline (L-CIT) have been investigated as precursors of NO to improve vascular function and blood pressure [26,27,28]. L-ARG added to a high cholesterol diet increased endothelial-dependent dilation or aortic rings in rabbits with hypercholesterolemia [29]. Similarly, short term and acute L-ARG supplementation improved brachial artery FMD in older adults [30] and in patients with hypertension [27], two populations characterized by endothelial dysfunction. A recent meta-analysis of 22 randomized placebo-controlled trials demonstrated an anti-hypertensive effect of L-ARG supplementation [31]. Interestingly, L-ARG supplementation was more effective to reduce diastolic blood pressure (DBP) in women than men [31]. Recently, oral supplementation with L-CIT has received much interest as L-ARG precursor for NO synthesis. L-CIT has a greater absorption than L-ARG since is not catabolized by intestinal arginase [32]. Moreover, L-ARG supplementation is less efficient for increasing plasma L-ARG due to gastrointestinal and hepatic removal upon absorption [33]. L-CIT supplementation has shown to efficiently increase plasma L-ARG levels to a greater extent than a similar dose of L-ARG [34,35], increasing NO bioavailability [36]. L-CIT has demonstrated to improve endothelial function in porcine coronary artery [37] and brachial artery of patients with vasospastic angina [38] and reduce resting peripheral PWV and blood pressure in middle-aged adults [39,40,41,42,43]. Although L-CIT supplementation has demonstrated some beneficial vascular effects in postmenopausal women, its potential to improve endothelial function and aortic stiffness remains unknown. Evidence shows that a single 10 g dose of L-CIT is a better L-ARG precursor than a similar dose of L-ARG and the most appropriate for clinical use in older adults [34]. Considering these findings, we hypothesized that L-CIT supplementation may improve vascular function and aortic blood pressure in postmenopausal women with hypertension. The main purpose of this study was to investigate the effects of 4 weeks of L-CIT supplementation on endothelial function, aortic stiffness, and blood pressure in hypertensive postmenopausal women.

## 2. Materials and Methods

### 2.1. Participants

Sedentary postmenopausal women aged 50 to 74 years of age were recruited from the Lubbock community to participate in this study. Sedentary was defined as <120 min per week of structured exercise or physical activity at low to moderate intensity for at least 6 months prior to beginning this study. Participants were postmenopausal for at least 1 year, with a body mass index ≤40 kg/m^2^ and a resting systolic blood pressure (SBP) ≥130 mmHg for unmedicated hypertensive women. Exclusion criteria included CVD, renal/pulmonary/metabolic diseases, diabetes (types 1 or 2), started hormone replacement therapy within the last 6 months, and taking beta-blockers or more than one antihypertensive medication.

### 2.2. Study Protocol

Participants were asked to come to the laboratory following an overnight fast and refrained from medications and caffeine for ~12 h and from alcohol for at least 24 h. During the first visit, participants were provided verbal explanation of the study, and then asked to sign an informed consent form followed by a health and exercise history questionnaire. This study was approved by the Texas Tech University Institutional Review Board and registered in ClinicalTrials.gov under NCT05227781. 

Height and weight were measured to calculate body mass index (BMI). After a 20 min rest period in the supine position in a dimly lit, temperature-controlled room (~23 °C), brachial BP was measured using an automated BP monitor (HEM-705CP; Omron Healthcare, Vernon, Hill, IL, USA) taken at least twice or until there was a <5 mmHg difference in SBP [44]. 

### 2.3. Measurement of Brachial and Aortic Blood Pressure, and Vascular Function

All laboratory measurements were conducted in the morning between 6 a.m. and 10 a.m. All post-intervention measurements were performed 48 hours after their last supplement dose to prevent any acute vascular effects of L-CIT. Radial tonometry measurements were obtained from the left wrist to estimate aortic BP at rest. Brachial diastolic BP (DBP) and mean arterial pressure (MAP) were used to calibrate the radial waveforms that were captured in 10-s increments. The aortic pressure waveforms were derived using a generalized validated transfer function (SphygmoCor CPv, AtCor Medical, Sydney, Australia). PWV was estimated using wave pressure sensors placed on the left carotid and femoral arteries. Distance between the carotid to femoral (cfPWV) segment was measured using a segmometer. CfPWV was calculated by measuring the transit time of the feet of the pulse waves relative to the distance between the pulse sites. Two cfPWV measurements were taken and averaged, ensuring a ≤0.3 m/s difference between each reading.

Right brachial artery diameter and mean blood velocity were measured using Doppler ultrasound equipped with a high-resolution linear array transducer (GE Logiq S7, Boston, MA, USA), secured hands-free 2–3 cm proximal to the antecubital fossa. A rapid-inflating cuff (Hokanson E20, Hokanson, Bellevue, WA, USA) was positioned around the upper forearm. After baseline measurements were obtained, the cuff was rapidly inflated to 250 mmHg for 5 min. After cuff deflation, diameter and blood velocity were recorded during the re-perfusion period for 3 min. The entire 10 min procedure was recorded in high-definition using a live-capture device on an external computer. This recording was subsequently uploaded onto an edge-detection software (Cardiovascular Suite, Quipu, Italy) which calculated FMD as the post-occlusion change in vessel diameter relative to baseline vessel diameter multiplied by 100: FMD% = [(peak diameter − baseline diameter)/baseline diameter] × 100.

### 2.4. Venipuncture and Quantification of L-Arginine

A blood draw was conducted via normal venipuncture techniques. Briefly, a 21-gauge butterfly needle was used to draw ~10 mL of blood into a serum separator tube, which was inverted 7–8 times and kept at room temperature (23 °C) for 30–45 min. The blood samples were then centrifuged for 10 min at 1000× *g*, aliquoted and stored in −80 °C for subsequent analysis of serum L-ARG. L-ARG was quantified using a commercially available colorimetric assay kit using company guidelines (Sigma-Aldrich, St. Louis, MO, USA).

### 2.5. L-CIT Supplementation

This study was a double-blind, placebo (PL) controlled parallel design. All participants and technicians were blinded from the supplements. Randomization was stratified by age and brachial SBP into a L-CIT or PL (maltodextrin) group. After baseline measurements, participants were given a 4-week supply of their respective supplement and asked to take 6 capsules in the morning and 7 at night, equating to 10 g/day. Participants were asked to bring their unused capsules back to the vascular health lab on their 4-week visit to calculate compliance to the supplement. Bi-weekly phone calls were made to ensure that participants were consuming the supplements in an appropriate dose. 

### 2.6. Statistical Analysis 

Power analysis was performed using G*Power, (version 3.0.10, Dusseldorf, Germany) using an alpha level set to 0.05. A priori power calculation determined that 8 subjects per group would be needed to observe an improvement of 3.05% FMD with a power of 86%, resulting in the need to recruit a minimum of 16 participants [30]. Normality of data was assessed using the Shapiro–Wilk test. One-way analysis of variance was used to detect potential differences between groups at baseline. A two-way repeated-measures analysis of variance with Bonferroni adjustments was used to determine possible differences between the groups (L-CIT vs. PL) over time (0 to 4 weeks) followed by appropriate post hoc tests when significant group-by-time interactions were observed. Statistical significance was set at *p* < 0.05. SPSS 26.0 was used to run all statistical analyses. 

## 3. Results

**Figure 1:**
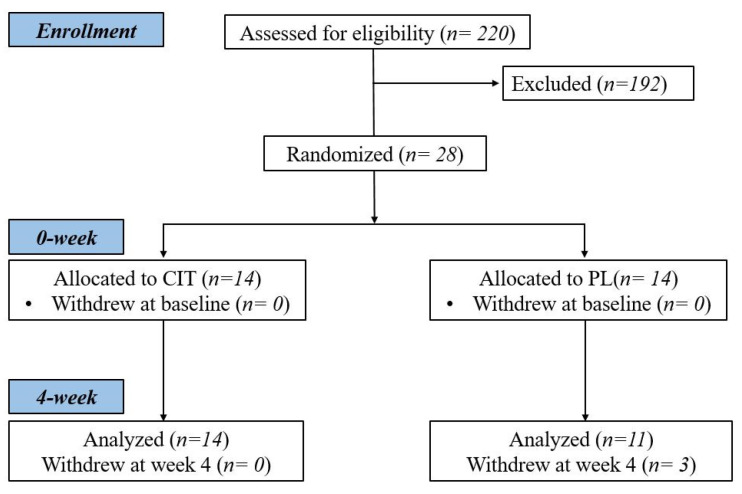
Study flow chart.

Twenty-eight women were randomized to CIT or PL groups, and 14 and 11 participants completed their supplementations, respectively (Figure 1). Participant characteristics and medications are reported in Table 1. There were no differences between the groups in age, height, weight, or BMI at baseline (all *p* > 0.05). Regarding compliance, participants consumed 92 ± 7% (L-CIT group) and 93 ± 7% (PL group) of the supplements.

Serum L-ARG levels, brachial artery characteristics, cfPWV, heart rate, and resting BP at baseline (0 week) and after 4 weeks of L-CIT or PL supplementation are reported in Table 2. There were no between-group differences in all measures at baseline.

**Figure 2:**
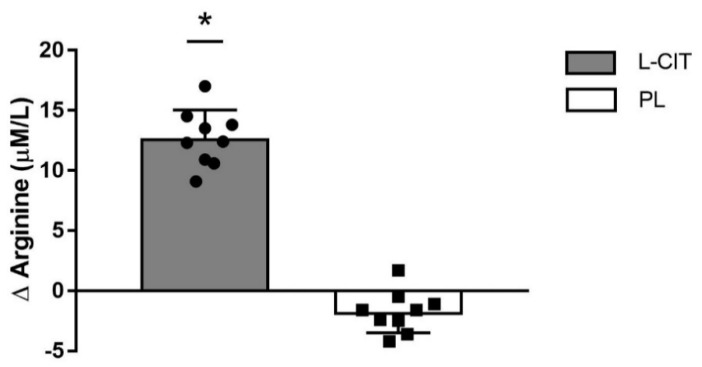
Individual data and group mean changes (Δ) in serum L−Arginine concentrations from 0 to 4 weeks of L-citrulline (L−CIT) and placebo (PL) supplementation in hypertensive postmenopausal women. * *p* < 0.01 vs. PL. For both groups, *n* = 9.

**Figure 3:**
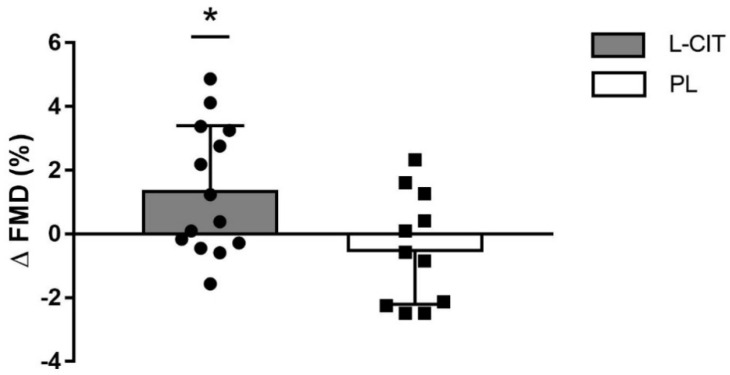
Individual data and group mean changes (Δ) in brachial artery flow−mediated dilation (FMD) from 0 weeks to 4 weeks of L-citrulline (L−CIT) and placebo (PL) in hypertensive postmenopausal women. * *p* < 0.05 vs. PL.

There were significant group-by-time interactions for serum L-ARG (*p <* 0.01), FMD (*p <* 0.05), and aortic DBP and MAP (*p <* 0.05 for both pressures). The changes (Δ) in serum L-ARG levels from 0 to 4 weeks were greater after L-CIT supplementation (12.7 ± 2.4 μM/L) compared to PL (−1.8 ± 1.7 μM/L, *p <* 0.01) (Figure 2). Baseline or peak brachial artery diameter and shear rate were not affected by L-CIT. L-CIT supplementation significantly increased FMD (1.4 ± 2.0%) compared to baseline (*p <* 0.05) and PL (−0.5 ± 1.7%) (Figure 3).

Both groups had no significant changes in aortic stiffness (cfPWV). However, the decrease in cfPWV after L-CIT trended to be significant (*p* = 0.07). 

**Figure 4:**
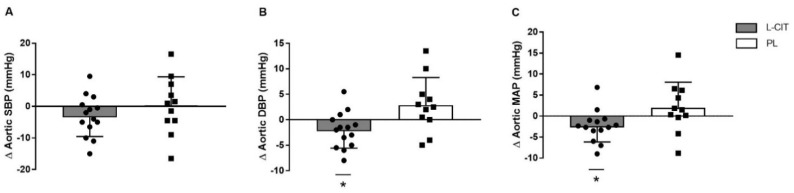
Individual data and group mean changes (Δ) in resting aortic systolic blood pressure (SBP, (**A**)), diastolic BP (DBP, (**B**)) and mean arterial pressure (MAP, (**C**)) from 0 weeks to 4 weeks of L−citrulline (L−CIT) and placebo (PL) in hypertensive postmenopausal women. * *p* ≤ 0.05 vs. PL.

Table 2 shows resting brachial and aortic pressures. There were significant group-by-time interactions for changes in (Δ) aortic ΔDBP and ΔMAP (*p* > 0.05 for both). Figure 4 presents aortic ΔSBP, ΔDBP, and ΔMAP from 0 to 4 weeks of supplementations. The change in aortic SBP with L-CIT supplementation (−3 ± 6 mmHg) was not significant different than PL (0.3 ± 9 mmHg). L-CIT supplementation significantly decreased aortic DBP (−2 ± 3 mmHg, *p* = 0.05) and MAP (−2 ± 3 mmHg, *p* ≤ 0.05) while no significant changes occurred after PL (aortic DBP: 3 ± 5 mmHg and aortic MAP: 2 ± 6 mmHg). There were no significant group-by-time interactions for any brachial pressures, nor for aortic SBP. 

## 4. Discussion

Findings from this study suggest that 4 weeks of L-CIT supplementation was effective for improving serum L-ARG levels, FMD and aortic DBP and MAP compared to PL in sedentary hypertensive postmenopausal women. CfPWV and brachial BP were not improved by 4 weeks of L-CIT. Taken together, 4 weeks of L-CIT seems to improve endothelial function and aortic DBP and MAP via increased L-ARG bioavailability in hypertensive postmenopausal women.

Most of the circulating L-ARG comes from the diet and less from the de novo production from L-CIT in the kidneys [45]. L-ARG is the substrate for the enzymes endothelial NO synthase and arginase and competes with methylarginines (e.g., asymmetric dimethyl-L-arginine (ADMA) and N-monomethyl-L-arginine) for binding to endothelial NO synthase [45]. Aging and menopause related increases in methylarginines production and arginase activity may contribute to the relative L-ARG deficiency and endothelial dysfunction [10,45,46]. Recent evidence of lower plasma L-ARG in women than men suggests a sex difference in endogenous L-ARG production [47]. 

In the present study, plasma L-ARG values at baseline were similar to the lowest values recently reported in obese women (61 μmol/L) [47]. As expected, serum L-ARG level was increased by L-CIT supplementation after 4 weeks (Figure 1). The relationship between increased circulating L-ARG concentrations and NO levels is contradictory. Although L-ARG concentrations can vary with L-ARG or L-CIT supplementation, elevated L-ARG may not increase NO levels [48]. However, evidence shows an increase in serum nitrate or urinary nitrate excretion with increased L-ARG concentration following L-CIT or ARG supplementation, implying that NO bioavailability can indeed be increased by elevated circulating L-ARG [35,49,50,51]. L-ARG availability for NO production is assessed by the L-ARG/ADMA ratio. The observed improvement in endothelial function following CIT supplementation has been exclusively attributed to the increase in the de novo L-ARG production as ADMA levels do not decrease [38,51]. Plasma L-ARG levels were increased after 4 weeks of L-ARG supplementation; however, it failed to increase FMD in healthy postmenopausal women [28]. In contrast, a 2-week L-ARG supplementation (16 g daily) increased FMD in older adults with age-related endothelial dysfunction [30]. A meta-analysis of 13 clinical trials including patients with cardiovascular diseases and cardio-metabolic risk factors concluded that short term L-ARG supplementation (3 to 21 g/day) was effective to increase FMD in individuals with an FMD value lower than 7% before the supplementation [26]. However, the high dose (>15 g daily) of L-ARG used in the previous studies may cause side effects including abdominal pain and osmotic diarrhea [52]. 

Although the current study did not quantify circulating NO and ADMA levels, it can be speculated that increased serum L-ARG had a positive effect on endothelial vasodilatory function, shown as an increase in FMD after 4 weeks of L-CIT supplementation (Figure 2). A factor that may have influenced the positive effect of L-CIT supplementation is the low baseline FMD in the L-CIT group (4.8 ± 2.1%). Conversely, acute ingestion of 10 g of L-CIT, the maximal clinically effective dose, did not improve microvascular reactive hyperemic blood flow in healthy older men [53] and older adults with heart failure [54]. Moreover, we found that an acute 6 g dose of L-CIT was unable to improve superficial femoral artery blood flow or vascular conductance during low-intensity exercise [55]. These previous findings suggest that L-CIT may need to be supplemented for at least 4 weeks to observe significant benefits in endothelial function. Morita and colleagues supplemented with L-CIT for 8 weeks at 800 mg/day in patients with vasospastic angina and observed improvements in FMD after 4 weeks, comparable to the present study [38]. L-CIT supplementation for 4 weeks can improve endothelial function, assessed by increased NO levels, via a decrease in arginase activity [36]. Arginase inhibition improves L-ARG availability for NO production by reducing the catabolism of L-ARG to ornithine [10,36]. This was one of the mechanism proposed for the decrease in FMD associated with menopause [10]. To our knowledge, this is the first study to report that L-CIT supplementation improves endothelial function (FMD) in hypertensive, otherwise healthy postmenopausal women, and sheds light on the potential for improving vascular function via a dietary intervention.

The association between endothelial dysfunction and the development of hypertension is well-documented [56]. The decrease in FMD begins during the early perimenopausal stage and progresses to values below 6% in healthy early postmenopausal women [8,10]. Evidence suggests that a faster rate of decline in FMD [9] and steeper increase in PWV [57] after the menopause contribute to greater cardiovascular risk in older women than men. Age-related increase in arterial stiffness causes systolic hypertension [57]. However, greater proximal aortic stiffening increases aortic blood pressure more in postmenopausal women than older men but without sex-related difference in brachial blood pressure [5]. It is recognized that aortic blood pressure relates to cardiovascular disease to a greater degree than brachial blood pressure [58]. Chronic hypertension causes ventricular wall hypertrophy, preventing the left ventricle from appropriately filling during diastole leading to heart failure with preserved ejection fraction, a more prevalent cardiovascular disease in older women than men [59]. Management of heart failure with preserved ejection fraction has surrounded treatment of SBP, but recommendations on the optimal DBP range has been largely ignored. This is important to consider, since coronary perfusion is dependent on aortic DBP, and coronary microvascular dysfunction [60] is apparent in heart failure patients [61,62]. An important analysis conducted by Sandesara et al. [63] found that a DBP ≥90 and <60 mmHg is associated with significant adverse risk in heart failure patients, providing a clinical target range for DBP. In the current study, a reduction in aortic DBP was detected after L-CIT supplementation compared to PL (Figure 4B). In agreement with our finding, a meta-analysis found that L-ARG supplementation decreases DBP by 2 mmHg in women but not in men [31]. Such decrease in DBP would reduce the prevalence of hypertension by 17% and the risk of heart failure by 6% [64]. The current study found that 4 weeks of L-CIT supplementation reduced DBP to more optimal levels (~80 mmHg) in hypertensive postmenopausal women.

DBP has shown to be influenced by systemic vascular resistance [65] and is exaggerated in hypertensive older adults during sympathetic activation [66]. Indeed, we found that watermelon powder containing 4 g/day of L-CIT and 2 g/day L-ARG supplemented for 6 weeks reduced brachial and aortic SBP and DBP at rest and during sympathetic activation [67]. Mechanistically, these findings suggest that L-CIT supplementation may have mitigated peripheral vasoconstriction resulting from increased sympathetic nerve activity. NO has shown a sympathoinhibitory effect in mice [68,69], which may attenuate the effect of sympathetic-mediated vasoconstriction. Previous investigations have seen L-CIT supplementation significantly lowering systemic arterial stiffness in middle-aged men [51] and women [43] with increased baseline values, which was attributed to improved endothelial function. These findings open two possible avenues for the efficacy of L-CIT to improve vascular function; improve endothelial function leading to increase NO bioavailability, which may attenuate exaggerated resting sympathetic activity in middle-aged and older adults [43,51]. Although systemic arterial stiffness is largely determined by the aorta, it is also influenced by the limb arteries. These findings are partially in line with results from this study, as we did see improvements in brachial artery endothelial function after L-CIT supplementation. Moreover, L-CIT reduced aortic stiffness (cfPWV) by ~0.66 m/s, although not statistically significant (*p* = 0.07). Our finding suggests that L-CIT may reduce cfPWV if supplemented for longer than 4 weeks. This reduction in cfPWV may protect women with hypertension from the 12% increased risk of cardiovascular events associated with each 1 m/s increase in systemic arterial stiffness [70].

Similar to our findings, a meta-analysis of 11 trials using L-ARG supplementation (median dose of 9 g/day and duration of 4 weeks) found an average of 5 mmHg and 3 mmHg decrease in brachial SBP and DBP, respectively [71]. In this current study, aortic MAP was reduced after L-CIT compared to PL (Figure 3C). There are two components of blood pressure throughout the arterial tree; pulsatile (pulse pressure), which represents the oscillatory component, and steady (MAP), which regulates organ perfusion [72]. Since MAP is calculated using SBP and DBP, it provides risk-related information associated with both pressures. In fact, a 1 million-participant meta-analysis showed that MAP was a more sensitive predictor of vascular mortality than SBP, DBP or pulse pressure [73]. Although our participants were still classified as having stage 2 hypertensive MAP [74], this significant reduction in aortic MAP with L-CIT was seen after only 4 weeks of supplementation. It can then be hypothesized that a longer intervention may evoke further reductions in MAP. 

This study is not without limitations. A larger sample size would have been beneficial to strengthen the findings of this paper and should be considered in future studies. Although we informed participants not to make any changes to their diet, we did not keep track of their diet using food logs. Further, we did not quantify circulating NO, which would provide more validation to our findings. Considering the property of L-CIT on reducing oxidative stress, examining circulating markers of oxidative stress in response to the intervention would provide further insight on additional mechanisms by which endothelial function was improved. These women, although hypertensive, were otherwise healthy individuals with no underlying conditions. Future studies are needed to examine the effects of L-CIT supplementation in other cohorts with known endothelial dysfunction such as obese with cardiometabolic risk factors [75] and type 2 diabetic [76] populations. Lastly, a longer intervention (≥8 weeks) using the high dose of L-CIT implemented in this study (10 g/day) in a hypertensive population has never been examined and would, in theory, elicit a more robust improvement in both endothelial function and blood pressure.

## 5. Conclusions

Four weeks of L-CIT supplementation was able to improve brachial artery FMD, serum L-ARG levels, and aortic DBP and MAP in hypertensive postmenopausal women. Our findings suggest that L-CIT improves endothelial function and aortic BP via increased L-ARG availability for NO-mediated vasodilation. Oral L-CIT supplementation may be a viable therapeutic strategy to combat the vascular complications that become apparent in hypertensive postmenopausal women.

## Figures and Tables

**Table 1:** Participant characteristics and medications.

Characteristics	L-CIT (*n* = 14)	PL (*n* = 11)	*p*
Age (years)	61 ± 6	64 ± 6	0.23
Height (meters)	1.58 ± 0.06	1.57 ± 0.07	0.97
Weight (kg)	74 ± 10	75 ± 15	0.78
Body Mass Index (kg/m^2^)	29.9 ± 4.1	30.9 ± 5.5	0.60
Hormone replacement therapy, *n*			
Estrogen	5	2	
Progesterone	0	1	
Anti-hypertensive medications, *n*			
Diuretic	0	1	
ACE Inhibitor	2	0	
CA^2+^ Channel Blocker	1	2	
ANG II Receptor Blocker	4	1	
Unmedicated	7	6	

Values are mean ± SD. Abbreviations: ACE, angiotensin converting enzyme; Ca^2+^, calcium; ANG II, angiotensin II; L-CIT, L-citrulline; PL, placebo. *p*-values are between-group differences from independent *t*-test.

**Table 2:** Changes in L-arginine, vascular function, and blood pressures after 4 weeks of L-citrulline supplementation.

Measure	L-CIT		PL		
	0 Week	4 Week	0 Week	4 Week	p *
L-ARG (µmol/L) ^¥^	81 ± 9	93 ± 8 ^†,‡^	81 ± 3	79 ± 3	0.01
Baseline brachial diameter (mm)	3.7 ± 0.5	3.7 ± 0.4	3.6 ± 0.3	3.7 ± 0.4	0.41
Peak brachial diameter (mm)	3.9 ± 0.5	3.9 ± 0.4	3.8 ± 0.3	3.9 ± 0.4	0.52
Baseline shear rate (s^−1^)	122 ± 37	124 ± 44	150 ± 56	146 ± 53	0.80
Peak shear rate (s^−1^)	1039 ± 428	1082 ± 455	1075 ± 267	1100 ± 323	0.91
FMD (%)	4.8 ± 2.1	6.2 ± 2.2 ^†,^*	4.7 ± 1.8	4.3 ± 1.7	0.03
cfPWV (m/s)	9.1 ± 2	8.5 ± 1.1	9.9 ± 1.2	9.3 ± 1.4	0.83
Heart Rate (beats/min)	64 ± 5	63 ± 6	63 ± 9	62 ± 8	0.65
Brachial Pressures					
SBP (mmHg)	139 ± 17	135 ± 17	136 ± 14	139 ± 14	0.30
DBP (mmHg)	83 ± 9	81 ± 8	79 ± 11	79 ± 14	0.47
MAP (mmHg)	101 ± 11	99 ± 10	98 ± 10	99 ± 13	0.60
Aortic Pressures					
SBP (mmHg)	126 ± 15	123 ± 11	127 ± 12	127 ± 14	0.29
DBP (mmHg)	84 ± 8	82 ± 8^†^	78 ± 11	81 ± 13	0.01
MAP (mmHg)	98 ± 9	96 ± 8^†^	94 ± 10	96 ± 13	0.01

Data are mean ± SD. Abbreviations: L-CIT, L-citrulline; PL, placebo; L-ARG, serum L-arginine; FMD, brachial artery flow mediated dilation; cfPWV, carotid-femoral pulse wave velocity; SBP, systolic blood pressure; DBP, diastolic blood pressure; MAP, mean arterial pressure. *p*-values are time-by-group interaction from two-way repeated measures ANOVA. **^¥^** *n* = 9 for both groups. * *p* < 0.05 vs. placebo; ^‡^ *p* < 0.01 vs. placebo; ^†^ *p* < 0.05 vs. 0 week.

## Data Availability

The data presented in this study are available upon request from the corresponding author.
